# The impact of obesity and overweight on response to internet-delivered cognitive behavioural therapy for adults with chronic health conditions

**DOI:** 10.1038/s41366-023-01285-6

**Published:** 2023-03-03

**Authors:** Andreea I. Heriseanu, Eyal Karin, Jennie Walker, Amelia J. Scott, Madelyne A. Bisby, Milena Gandy, Joanne Dudeney, Alana Fisher, Nickolai Titov, Blake F. Dear

**Affiliations:** 1 eCentreClinic, School of Psychological Sciences, Macquarie University, Sydney, NSW Australia; 2 School of Psychological Sciences, Macquarie University, Sydney, NSW Australia; 3 MindSpot Clinic, MQ Health, Macquarie University, Sydney, NSW Australia

**Keywords:** Health care, Medical research

## Abstract

**Background:**

There is growing evidence that internet-delivered cognitive behavioural therapy (iCBT) can improve functioning and reduce psychological distress in people with chronic health conditions. Obesity frequently co-occurs with chronic health conditions, yet its impact on response to psychological interventions in this population is not known. The current study examined associations between BMI and clinical outcomes (depression, anxiety, disability, and satisfaction with life) following a transdiagnostic iCBT program targeting adjustment to chronic illness.

**Methods:**

Participants from a large randomised controlled trial, who provided information on height and weight, were included (*N* = 234; mean age= 48.32, SD = 13.80; mean BMI = 30.43, SD = 8.30, range 16.18–67.52; 86.8% female). The influence of baseline BMI range on treatment outcomes at post-treatment and 3-month follow-up was examined using generalized estimating equations. We also examined changes in BMI and in participants’ perceived impact of weight on their health.

**Results:**

Improvement in all outcomes occurred across BMI ranges; additionally, persons with obesity or overweight generally experienced greater symptom reductions than those within a healthy weight range. A greater proportion of participants with obesity achieved clinically significant change on key outcomes (e.g., depression: 32% [95% CI: 25%, 39%]) than participants with a healthy weight (21% [95% CI: 15%, 26%]) or overweight (24% [95% CI: 18%, 29%], *p* = 0.016). There were no significant changes in BMI from pre-treatment to 3-month follow-up, however there were significant reductions on the self-rated impact of weight on health.

**Conclusions:**

Persons with chronic health conditions and with obesity or overweight benefit at least as much as those with a healthy BMI from iCBT programs targeting psychological adjustment to chronic illness, even without changes in BMI. iCBT programs may be an important component in the self-management of this population, and may address barriers implicated in health behaviour change.

## Introduction

Chronic health conditions, characterised by their long-lasting and persistent effects, are a substantial cause of disability and disease burden [1], with increasing global prevalence [2]. Several common chronic health conditions are associated with increased prevalence of obesity, including type 2 diabetes, cardiovascular diseases, and musculoskeletal pain conditions, such as osteoarthritis [3]. Obesity may represent an aggravating or triggering factor for other chronic health conditions (e.g. in rheumatic diseases), while concurrently, health conditions such as chronic pain may aid the development of obesity, through physical inactivity or the use of eating behaviour for rewarding or analgesic effects [3, 4]. Multi-morbidity, where two or more conditions co-occur, is one of the greatest challenges currently facing health systems and requires integrated treatment approaches and self-management [5]. Approximately 30–50% of persons with chronic health conditions report difficulties with adjustment, distress and disability, especially in the context of multi-morbidity [1, 6], whilst rates of depression and anxiety are higher amongst persons with chronic health conditions than in the general population [7, 8]. Higher rates of anxiety and depression are also present in persons with obesity compared to those in the healthy weight range, with reciprocal links existing between depression and obesity [9, 10]. Chronic health conditions and obesity [11–13] have also been associated with higher self-reported disability and lower life satisfaction. Therefore, complex interactions exist between high weight, the presence of chronic health conditions, and poorer mental health and subjective well-being. Better management of chronic conditions to improve health outcomes, including better understanding the mechanisms and contributors to adjustment and distress, is a key health system priority [14].

There is growing evidence that psychological treatment, especially cognitive behavioural therapy (CBT) can promote adjustment to illness in people with chronic health conditions, such as improvements in functioning and psychological wellbeing [15]. A recent review of face-to-face CBT for chronic health conditions has found significant, moderate improvements in depression and anxiety [16]. While face-to-face treatment presents barriers such as cost, impaired physical access due to mobility issues, and limited availability of adequately trained local clinicians, internet-delivered approaches may reduce the impact of many of these barriers, thereby presenting a promising avenue for improving self-management and mental health amongst persons with chronic illness. A recent review of internet-delivered CBT (iCBT) for individuals with health conditions demonstrated small to moderate improvements in depression and anxiety symptomatology (anxiety: SDM = 0.45 ± 0.09, *p* < 0.001 at post-treatment and SDM = 0.51 ± 0.03, *p* < 0.001 at 3-month follow-up; depression: SDM = 0.31 ± 0.04, *p* < 0.001 at post-treatment and SDM = 0.66 ± 0.08, *p* < 0.001 at 3-month follow-up) [17].

Less is known about the effects of psychological treatment, including both face-to-face CBT and iCBT, in people with co-occurring chronic health conditions and high weight (i.e. obesity or overweight), despite their frequent overlap, and their common clustering with poor mental health. In a multidisciplinary pain rehabilitation program with a considerable psychological component, program participants with severe obesity (BMI ≥ 35) showed the least improvement in health-related quality of life, although they showed similar improvements in anxiety and depression to those within lower weight ranges [18]. A recent review [4] found that obesity had a negative impact on functioning and pain outcomes in people with psoriatic arthritis, and data from three RCTs found a similar detrimental impact in osteoarthritis [19]. An RCT of behavioural and psychological interventions for combined depression and obesity found reductions in depression severity and body weight that were not clinically significant [20]. Cross-sectionally, an association exists between increasing BMI and depression, disability and quality of life in patients with chronic pain [21, 22] Persons with chronic fatigue syndrome and with overweight or obesity have significantly worse physical and mental health related quality of life, more severe fatigue, and greater disability [23]. In addition, people with obesity show lower response rates to antidepressant treatment compared to those without obesity [24, 25]. Given these findings, it is possible that increased weight may complicate treatment aimed at improving mental health, functioning and quality of life in persons with chronic health conditions.

The current study sought to examine the associations between BMI range and the clinical outcomes of a randomised controlled trial (RCT) of an iCBT program aiming to promote adjustment to illness in adults with chronic health conditions, based on cognitive behavioural therapy principles (the Chronic Conditions Course [26]). This study used a two-arm RCT design comparing the iCBT-based program versus waitlist control, with control participants crossing over to the iCBT-based program at the conclusion of the 8-week waitlist phase, and completing the same measures at the same time points as the participants in the treatment group.

In this program, the treatment group reported statistically and clinically significant improvements in the primary outcomes (depression, anxiety, and disability) compared to a treatment-as-usual control group, with high completion rates and level of satisfaction. In the current study, secondary analyses examined associations between baseline BMI range and treatment response at post-treatment and 3-month follow-up, as well as changes in weight and self-rated impact of weight on health between pre-treatment and 3-month follow-up. Given some evidence of reduced response to treatment in people with obesity, it was hypothesised that (1) higher BMI range at baseline would predict poorer treatment response for depression, anxiety, self-reported disability and life satisfaction at post-treatment and at 3-month follow-up, and that (2) BMI and its self-reported health impact would not shift between pre-treatment and 3-month follow-up, due to the fact that the intervention did not specifically target factors generally considered to be implicated in the maintenance of obesity and overweight (such as diet, formal instructions for exercise, shape and weight concerns, and disordered eating).

## Methods

### Participants

The current study used a subsample of participants (*N* = 234) from a previous large RCT (*N* = 676) who provided height and weight information at pre-treatment and 3-month follow-up [26]. This data was only collected in the later phases of the trial, following feedback from trial participants in earlier phases, indicating that weight may be contributing to their adjustment difficulties. Inclusion criteria for the trial were (1) aged 18 years or older, (2) living in Australia, (3) self-reported a diagnosis of a chronic physical health condition, and (4) self-reported that their health condition impacted their emotional wellbeing and quality of life. Participants were excluded if they reported very severe symptoms of depression (>24 on the PHQ-9) or if they reported acute suicidality or a recent history of attempted suicide.

### Design

This study used a single-arm, retrospective design. All participants included in this secondary analysis received the intervention, and had data collected at the same time-points, hence data across the two arms of the original RCT was collapsed to enable within-group comparisons.

### Intervention

The Chronic Conditions Course is a remotely delivered intervention aiming to promote adjustment to chronic health conditions. It is based on CBT and transdiagnostic principles and was designed to deliver psychoeducation and self-management skills to adults with a broad range of health conditions. It consisted of five core modules delivered sequentially across eight weeks, with recommended home-based tasks, case stories, and additional resources also provided (e.g. managing sleep, challenging beliefs). Modules were presented in slideshow format via the eCentreClinic software platform and required approximately 20 min to complete. In brief, the modules covered psychoeducation on chronic health conditions and psychological adjustment, cognitive-behavioural formulation, cognitive challenging, behavioural activation, controlled breathing, activity pacing, graded exposure, goal-setting and managing health lapses. Participants also had individual weekly contact with a study clinician (a registered psychologist or clinical psychologist) for 8 weeks via telephone or secure messaging. The primary outcome measures of the trial were depression symptoms, anxiety symptoms and disability; overall life satisfaction constituted a secondary outcome. For a comprehensive description of the program, including its the structure and content, see Dear et al. [26].

### Measures

#### Anthropometric measurement

Participants were asked to self-report their height in cm and their weight in kg (a method considered to be adequate for remotely collected data [27]). BMI was computed as kg/m^2^ and was categorised into three ranges: underweight and healthy weight (BMI < 25; these two ranges were combined as only three participants (~1%) of the sample had a BMI in the “underweight” range, i.e. <18.5), overweight (25≤ BMI < 30), and obesity (BMI ≥ 30).

#### Weight impact

A single question was asked regarding perceived impact of weight on health: “Is your weight an issue for you in terms of your physical or mental health?” with “No”, “Yes” and “Unsure” as possible responses.

#### Depression symptoms

This was assessed using the Patient Health Questionnaire 9-item (PHQ-9) [28], a widely used and validated measure, including in samples with chronic health conditions [29]. Scores range from 0 to 27 with higher scores indicating higher depression severity.

#### Anxiety symptoms

Anxiety symptoms were assessed using the Generalized Anxiety Disorder 7-item (GAD-7) [30], which has also demonstrated adequate psychometric properties in medical populations [31]. Scores range from 0 to 21, with higher scores indicating higher anxiety severity.

#### Disability

The 12-item World Health Organization Disability Assessment Schedule 2.0 (WHODAS-12) [32], designed and validated for use in patients with health conditions [33], was used for assessing disability. It provides a measure of functioning across six domains (communication, self-care, mobility, interpersonal relationships, life activities, and community participation). Scores range from 0 to 48, with higher scores indicating greater disability.

#### Satisfaction with Life

Overall life satisfaction was assessed using the 5-item Satisfaction with Life Scale (SWLS) [34]. Scores on the SWLS range from 5 to 35, where higher scores indicate greater life satisfaction.

#### Treatment characteristics

Adherence and treatment satisfaction were also assessed at post-treatment. Satisfaction was measured using a purpose-built questionnaire with responses ranging from 1 - “very satisfied” to 5 - “very unsatisfied” (see Dear et. al [26]) and adherence was defined as the completion of ≥ 4 out of 5 modules.

#### Statistical analysis

Analyses were conducted using IBM SPSS v28. Descriptive statistics were calculated for baseline demographics and clinical characteristics using unweighted data. Differences in demographic and clinical characteristics by BMI range were tested using ANOVAs and chi-squared analyses. Multiple imputation was used to address missing data (11.7% at post-treatment, and 16.3% at 3-month follow-up); the model included baseline symptom severity, number of modules completed, and BMI [35]. Alpha was set to 0.05 for all analyses.

To examine the effect of BMI categories on clinical outcomes, marginal models were employed. The model was fit using generalised estimating equations (GEE) with a gamma distribution and a log-link function. An unstructured working correlation matrix with robust standard errors were specified [36, 37]. Time, BMI range, and their interaction were entered as predictors. GEE analyses were conducted using weighted data. Frequency weighting, determined by population proportion of BMI ranges [38], was applied to the data to create a balanced design and account for under-sampling of the healthy weight range and oversampling of the obesity range. This approach is consistent with epidemiological research and stratification [39]. Metrics of symptom change included percentage change (95% confidence intervals) and Cohen’s *d* (95% confidence intervals) from pre- to post-treatment, and from post-treatment to 3-month follow-up.

To examine clinically significant change, we reported the proportion of individuals who achieved a minimal treatment-related symptom improvement (≥30% reduction) [26, 40] or clinically significant improvement (≥50% reduction) in symptoms at post-treatment for each of the primary outcome measures. Logistic regression models tested clinical change for each outcome measure by BMI range and overall.

The longitudinal GEE models estimating symptom changes were also modelled to include available potential moderators of the effect of BMI range on symptom change. These models included the adjustment for a covariate with a time x covariate interaction (i.e. a three-way interaction between time, BMI range and moderator). The moderator variables explored included demographics (age, sex), treatment-related variables (satisfaction, adherence), as well as clinical and weight characteristics (baseline BMI, perception of weight as a problem, and number of chronic conditions). These analyses were conducted as sensitivity analyses (consistent with STROBE guidelines [41, 42]) and are included in Supplementary Table 1.

## Results

### Baseline demographic and clinical characteristics

Table 1 displays the baseline demographic and clinical characteristics of the study sample. No marked differences were observed in these characteristics between the three BMI categories, with the exception of age (the healthy weight group was, on average, younger than the other groups, *p* = 0.018). Chronic pain and myalgic encephalomyelitis/chronic fatigue syndrome (ME/CFS) were the most commonly endorsed primary health conditions.

**Table 1:** Baseline demographic and clinical characteristics and treatment characteristics.

	HW *n* = 66	OW *n* = 61	OB *n* = 107	p_between-group_	Overall (*n* = 234)
*Age (M, SD)*	44.26 (15.06)	49.87 (12.78)	49.95 (13.15)	0.018	48.32 (13.80)
*Sex (n, %)*					
Female	54 (81.8%)	55 (90.2%)	94 (87.9%)	0.345	203 (86.8%)
Male	12 (18.2%)	6 (9.8%)	13 (12.1%)		31 (13.2%)
*Employed (n, %)*	29 (43.9%)	24 (39.3%)	40 (37.4%)	0.691	93 (39.7%)
*Married/in a relationship (n, %)*	38 (57.6%)	38 (62.3%)	59 (55.1%)	0.665	135 (57.7%)
*Tertiary education (n, %)*	31 (47.0%)	27 (44.3%)	46 (44.2%)	0.877	104 (44.4%)
*No. chronic conditions (M, SD)*	3.52 (2.54)	3.23 (2.10)	3.85 (2.54)	0.271	3.59 (2.44)
*BMI range (n, %)*					
HW					66 (28.2%)
OW					61 (26.1%)
OB					107 (45.7%)
*Primary health condition (n, %)*					
Chronic Pain^*^	28 (42.4)	32 (52.5)	53 (49.5)		117 (50.0)
ME/CFS	11 (16.7)	5 (8.2)	9 (8.4)		25 (10.7)
Parkinson’s Disease	3 (4.5)	3 (4.9)	3 (2.8)		9 (3.8)
Cancer	2 (3.0)	3 (4.9)	3 (2.8)		8 (3.4)
Ehlers Danlos Syndrome	3 (4.5)	3 (4.9)	2 (1.9)		8 (3.4)
MS	1 (1.5)	2 (3.3)	3 (2.8)		6 (2.6)
Scleroderma	2 (3.0)	0 (0.0)	3 (2.8)		5 (2.1)
Lupus	2 (3.0)	0 (0.0)	3 (1.9)		4 (1.7)
Other	22 (33.3)	13 (21.3)	42 (39.3)		78 (33.3)

^*^Chronic pain included the following conditions: all arthritis types, fibromyalgia, neuropathic pain, endometriosis, headache/migraine, temporomandibular joint dysfunction, and musculoskeletal pain conditions. *HW* healthy weight BMI range, *OW* overweight BMI range, *OB* obesity BMI range. Participant *n*s are unweighted.

### Treatment characteristics

There were no significant differences between the BMI groups in terms of number of modules completed (HW *M*(*SE*) = 4.38 (0.07), OW *M*(*SE*) = 4.38 (0.07), OB *M*(*SE*) = 4.24 (0.09), *p* = 0.384) and treatment adherence (HW *M*(*SE*) = 80% (3.0%), OW *M*(*SE*) = 82% (3.0%), OB *M*(*SE*) = 81% (3.0%), *p* = 0.893). A significant difference for satisfaction with treatment was observed between the three BMI groups, where the group with obesity endorsed slightly lower satisfaction with the treatment than the other groups (HW *M*(*SE*) = 1.74 (0.06), OW *M*(*SE*) = 1.67 (0.05), OB *M*(*SE*) = 1.48 (0.05), *p* < 0.001).

### BMI and self-reported impact on health

Mean BMI at baseline was 30.43 (SD = 8.30, range 16.18–67.52), i.e. within the “obesity” range, which constituted the most frequent BMI range in the study (*n* = 107, 45.7%; healthy weight/underweight *n* = 66, 28.2%; overweight *n* = 61, 26.1%). BMI did not significantly change from pre-treatment to 3-month follow-up (*p* = 0.821; see Table 2). Most participants considered their weight to be impacting their physical and/or mental health. At baseline, 90% and 54% of the participants with obesity and overweight, respectively, perceived their weight to be negatively impacting their health, compared to approximately 30% of those with a BMI in the healthy range. At 3-month follow-up, these numbers decreased to 85% (obesity), 50% (overweight) and 28% (healthy weight). The perception of weight as a problem significantly differed between the BMI ranges as described above, such that with increasing BMI, there was an increasing proportion of participants perceiving their weight to be a problem for their health at both time points (*p* < 0.001); furthermore, a significant decrease in the perception of weight as a problem occurred between the two time points (*p* < 0.001), however this decrease was relatively even across BMI ranges (i.e., the interaction of BMI and time did not reach significance, *p* = 0.288).

**Table 2:** Change in weight-related variables.

	*N*	Pre (EMM, 95%CI)			3MFU (EMM, 95%CI)			*p*_*pre to 3mfu*_
BMI^*^								
HW	66	22.11 (21.62, 22.60)			22.02 (21.47, 22.56)			
OW	61	27.35 (27.03, 27.67)			27.66 (27.11, 28.22)			
OB	107	37.32 (35.97, 38.68)			36.96 (35.49, 38.43)			
BMI x Time								0.178
Overall	234	30.43 (29.37, 31.50)			30.33 (29.25, 31.41)			0.821
		Pre (%)			3MFU (%)			
*Weight as health problem*		Yes	Unsure	No	Yes	Unsure	No	
HW	66	29.60%	20.70%	49.80%	27.60%	13.10%	59.30%	
OW	61	54.20%	16.70%	29.20%	50.00%	10.40%	39.60%	
OB	107	90.00%	4.70%	5.30%	85.30%	5.30%	9.50%	
BMI x Time								0.288
Overall	234	57.50%	14.10%	28.40%	53.90%	9.60%	36.50%	<0.001

*3MFU* 3-month follow-up, *EMM* estimated marginal mean, *HW* healthy weight BMI range, *OW* overweight BMI range, *OB* obesity BMI range. Participant *n*s are unweighted.

### BMI range and baseline measures

At baseline, although differences were small, a BMI gradient could be observed, such that participants with obesity had higher depression, anxiety and disability scores, and lower satisfaction with life than participants who were overweight, who, in turn, displayed the same pattern of differences from participants within a healthy BMI range (see Table 3). Although most of these differences did not reach significance, the group with obesity reported significantly higher baseline disability than the healthy-weight group (*p* = 0.004).

### BMI range and treatment outcomes

GEE analyses revealed a significant main effect of time across all outcomes, indicating significant improvement in symptoms between pre- and post-treatment, regardless of BMI range (*p* < 0.001; see Table 3). These changes were at 3-month follow-up. Significant BMI by time interactions were found from pre- to post-treatment for depression and disability, indicating that BMI influenced participants’ trajectory of improvement. Significant BMI by time interactions were also present for all outcomes from post-treatment to 3-month follow-up. Table 4 lists the percentage symptom changes. From pre- to post-treatment, participants in the overweight or obesity range had a significantly higher percentage of symptom change than those in the healthy weight group for disability (*p* = 0.029), and participants with obesity had an equivalent percentage of symptom change for depression to those in the healthy weight range, which was higher than in those with overweight (*p* = 0.016). Between post-treatment and 3-month follow-up, participants with overweight or obesity generally had a higher percentage of symptom change than those with healthy weight across outcomes (all *p*s < 0.05). Furthermore, the proportion of participants with clinically significant improvement at post-treatment (i.e. ≥50% symptom reduction from baseline) significantly differed by BMI range, such that a higher proportion of persons with obesity reached this threshold for depression (*p* = 0.016) and disability (*p* = 0.034), and with more participants in the overweight and obesity ranges reaching this threshold for satisfaction with life (*p* = 0.007) (see Table 4).

**Table 3:** Estimated marginal means and percentage improvement in clinical outcomes.

	*N* (weighted)	Estimated marginal means (SE)					Percentage change (95% CI)		*d*_within-group_ (95% CI)	
		Pre	Post	*p*_*pre to post*_	3MFU	*p*_post to 3mfu_	Pre→Post	Post→3MFU	Pre→Post	Post→3MFU
*Depression*										
HW	239	11.91 (0.28)	8.71 (0.31)		9.75 (0.41)		27 (22, 32)	−12 (−22, −2)	0.68 (0.49, 0.87)	0.18 (0.03, 0.32)
OW	239	12.07 (0.34)	9.80 (0.43)		8.88 (0.41)		19 (12, 26)	9 (1, 18)	0.40 (0.21, 0.58)	0.18 (0.04, 0.32)
OB	239	12.73 (0.35)	9.35 (0.44)		9.65 (0.44)		27 (20, 33)	−3 (−12, 6)	0.60 (0.41, 0.79)	0.06 (−0.09, 0.2)
BMI x Time				0.016		<0.001				
Overall	717	12.18 (0.19)	9.26 (0.23)	<0.001	9.41 (0.24)	0.449	24 (28, 20)	−2 (−7, 3)		
*Anxiety*										
HW	239	8.69 (0.29)	6.69 (0.31)		7.28 (0.40)		23 (16, 30)	−9 (−21, 3)	0.43 (0.24, 0.61)	0.11 (−0.03, 0.25)
OW	239	9.04 (0.35)	6.73 (0.40)		6.07 (0.47)		26 (17, 34)	10 (−6, 25)	0.46 (0.28, 0.65)	0.15 (0.01, 0.29)
OB	239	9.15 (0.33)	6.88 (0.40)		7.02 (0.43)		25 (16, 33)	−2 (−15, 10)	0.43 (0.24, 0.61)	0.03 (−0.11, 0.17)
BMI x Time				0.659		0.038				
Overall	717	8.94 (0.19)	6.75 (0.22)	<0.001	6.79 (0.21)	0.549	24 (19, 29)	0 (−7, 6)		
*Disability*										
HW	239	21.46 (0.59)	19.08 (0.72)		18.48 (1.04)		11 (5, 18)	3 (−9, 15)	0.23 (0.05, 0.41)	0.07 (−0.07, 0.21)
OW	239	22.92 (0.58)	19.12 (0.68)		16.66 (0.65)		17 (11, 22)	13 (6, 20)	0.41 (0.22, 0.59)	0.26 (0.12, 0.40)
OB	239	24.00 (0.62)	19.34 (0.79)		18.61 (0.84)		19 (13, 26)	4 (−5, 12)	0.48 (0.29, 0.66)	0.07 (−0.07, 0.21)
BMI x Time				0.029		0.026				
Overall	717	22.66 (0.35)	19.16 (0.43)	<0.001	17.88 (0.53)	0.006	15 (12, 19)	7 (1, 13)		
*Satisfaction with life*										
HW	239	14.73 (0.43)	16.44 (0.50)		16.66 (0.59)		−12 (−18, −5)	−1 (−9, 6)	0.24 (0.06, 0.43)	0.04 (−0.1, 0.18)
OW	239	14.15 (0.46)	16.57 (0.56)		18.23 (0.66)		−17 (−25, −9)	−10 (−19, −1)	0.34 (0.16, 0.53)	0.24 (0.1, 0.38)
OB	239	13.86 (0.45)	16.61 (0.63)		16.01 (0.66)		−20 (−29, −11)	4 (−5, 12)	0.39 (0.20, 0.57)	0.08 (−0.06, 0.22)
BMI x Time				0.176		0.014				
Overall	717	14.25 (0.26)	16.53 (0.37)	<0.001	16.98 (0.37)	0.243	−16 (−22, −10)	−3 (−7, 2)		

*3MFU* 3-month follow-up, *HW* healthy weight BMI range, *OW* overweight BMI range, *OB* obesity BMI range. Measures used: depression – PHQ-9; anxiety – GAD-7; disability – WHODAS-12 2.0; satisfaction with life – SWLS. Participant ns are weighted.

**Table 4:** Clinically significant improvement.

	Pre-treatment to post-treatment						Pre-treatment to 3-month follow-up				
	BMI range			*p*_between_	Overall		BMI range			*p*_between_	Overall
	HW *n* = 239	OW *n* = 239	OB *n* = 239				HW *n* = 239	OW *n* = 239	OB *n* = 239		
*Depression*						*Depression*					
≥30%	41% (34, 48)	41% (34, 48)	47% (39, 55)	0.313	43% (38, 47)	≥30%	45% (37, 52)	52% (45, 60)	45% (37, 54)	0.208	47% (42, 52)
≥50%	21% (15, 26)	24% (18, 29)	32% (25, 39)	0.016	25% (21, 29)	≥50%	24% (18, 29)	31% (25, 37)	28% (21, 35)	0.180	27% (24, 31)
*Anxiety*						*Anxiety*					
≥30%	40% (33, 47)	48% (41, 55)	45% (36, 55)	0.159	44% (39, 49)	≥30%	43% (34, 53)	54% (45, 64)	47% (37, 56)	0.119	48% (44, 52)
≥50%	29% (22, 35)	30% (22, 38)	32% (25, 39)	0.547	30% (26, 34)	≥50%	33% (27, 39)	42% (34, 49)	37% (28, 45)	0.149	37% (33, 41)
*Disability*						*Disability*					
≥30%	28% (22, 34)	29% (22, 36)	37% (30, 44)	0.072	31% (27, 35)	≥30%	31% (19, 43)	52% (43, 60)	42% (32, 52)	<0.001	41% (34, 48)
≥50%	13% (9, 18)	12% (8, 16)	21% (14, 27)	0.034	15% (12, 18)	≥50%	19% (10, 29)	34% (28, 40)	21% (14, 28)	0.001	25% (21, 29)
*Satisfaction with life*						*Satisfaction with life*					
≥30%	32% (26, 39)	33% (26, 40)	38% (30, 45)	0.414	34% (30, 38)	≥30%	327 (29, 44)	52% (44, 60)	36% (27, 46)	0.002	42% (36, 48)
≥50%	15% (9, 20)	24% (17, 31)	27% (20, 35)	0.007	22% (18, 25)	≥50%	22% (14, 30)	43% (34, 52)	25% (18, 33)	<0.001	30% (24, 36)

*HRQoL* health-related quality of life, *HW* healthy weight BMI range, *OW* overweight BMI range, *OB* obesity BMI range. Measures used: depression – PHQ-9; anxiety – GAD-7; disability – WHODAS-12 2.0; satisfaction with life – SWLS. Participant *n*s are weighted.

Moderator analyses conducted with potential covariates revealed that perception of weight as a problem was the only variable which systematically and significantly affected the observed difference in rate of change between BMI ranges on the study outcomes. This variable appeared to impact the rate of change in the healthy weight group, such that participants within this group who considered their weight to be a problem for their health experienced a reduced rate of symptom improvement across all outcomes (for more information, see Supplementary Table 1).

## Discussion

The current study is the first to report the influence of BMI range on outcomes of an iCBT intervention targeting psychological adjustment to chronic health conditions. Contrary to our hypothesis, participants with obesity or overweight appeared to derive greater benefits from treatment, with a more favourable trajectory of change over the course of the intervention for most of the outcomes. In this way, despite starting with higher symptom severity at baseline, these participants’ post-treatment scores were comparable with those of the healthy weight group. Further, a higher proportion of participants with obesity reached clinically significant improvement across most outcomes.

One possibility for this finding is that iCBT provides additional benefit for persons with high weight by addressing particular issues present in this group, even if these are not specifically targeted by the intervention, or directly measured as an outcome. One such issue may be the reduction of internalised weight-based stigma, which is prevalent in persons with obesity [43], and which has been shown to be amenable to modification through CBT [44]. This may result in an increase in hope and self-efficacy, which are positively associated with symptom change [45].

At pre-treatment, most persons in the high weight groups perceived their weight to be detrimental to their physical and/or mental health. Participants’ perception of the relationship between their weight and their health shifted over time, such that fewer people saw their weight as negatively affecting their physical and/or mental health at 3-month follow-up. This could be a product of the intervention’s focus on adjustment to chronic health conditions (a model which could be applicable to obesity, which has been classified as a chronic, relapsing non-communicable disease by the European Commission, the American Medical Association, and other bodies [46]), rather than on ameliorating symptoms of the condition itself (i.e. weight, in the case of obesity). A key message of the present intervention is that one can enact health-promoting behaviours, and can achieve sound psychological health, despite the presence of physical health conditions, and it is possible that this message was generalised to the presence of high BMI, although this was not specifically targeted.

A surprisingly high number of people with a BMI within the healthy range (~30%) also perceived their weight as negatively impacting their health at pre-treatment. Therefore, another potential (or additional) explanation for the different rates of change in symptoms experienced according to BMI range is that this perception of weight as problematic in the healthy weight group may be driving a lower rate of change in this group, compared to the higher weight groups. Such a perception is likely unfounded in this group, and may be indicative of distorted cognitions regarding shape and weight, such as those present in eating disorders [47]. This is plausible, as eating disorders have significant associations with chronic physical health conditions, such as fibromyalgia, diabetes, and hypertension [48], and chronic health conditions - for example, pain [49] and gastrointestinal conditions [50] commonly present with disordered eating behaviours.

Almost half of participants in this treatment-seeking sample had a BMI in the “obesity” range. This is consistent with the body of research linking chronic pain, multi-morbidity and obesity [51]. BMI did not significantly change following the intervention. This is unsurprising, as factors directly implicated in weight loss such as eating patterns and formal exercise-based interventions were not targeted in this program. Previous research has shown that CBT that did not include behavioural weight loss principles improved depression symptoms, but did not lead to significant weight loss, in people with obesity and comorbid depression [52]. Nevertheless, CBT interventions represent a beneficial component in the health management in persons with obesity, as depression and anxiety are well-known barriers to lifestyle change interventions [53, 54], while the inclusion of cognitive and behavioural strategies has been shown to improve adherence to lifestyle interventions [55]. Further, there is some evidence that psychological interventions concurrently targeting physical health parameters (such as glycaemic management in diabetes) and chronic health condition-specific distress are effective [56]. Hence, future avenues for tandem interventions which introduce psychological adjustment and CBT principles alongside health behaviour change principles are promising.

This study included several limitations. First, we used categories based on BMI, which is an imperfect measure of both adiposity and health [57]. Another measure (such as waist circumference), a composite index, or a clinical staging system including multiple parameters (such as the Edmonton Obesity Staging System [58]) may have resulted in a more clinically meaningful categorisation of participants. However, height and weight are simple to measure, whether abdominal obesity measurements are more prone to measurement error (especially in those with obesity or overweight), even when performed by healthcare professionals [59]. Second, BMI and perception of weight data was only collected in later phases of the trial, which precluded the inclusion of all participants from the original trial, and only at pre-treatment and 3-month follow-up, due to participant burden considerations. Third, no information was collected on participant activities (e.g. any additional treatment) during the follow-up period. Fourth, this research did not include formal measures of weight stigma, eating disorder psychopathology, and self-efficacy, i.e. some of the variables hypothesised to be driving the observed differential trajectories of improvement according to weight. Future research aimed at elucidating the mechanisms underpinning the findings should also incorporate these, as well as any other relevant variables derived from qualitative interviews with participants. And finally, due to the absence of a control group, caution must be employed when ascribing the findings to the intervention, as it is possible for other factors to have contributed to the results.

In conclusion, the current study found that people with chronic health conditions, regardless of weight, benefitted from an iCBT intervention for psychological adjustment. Those with overweight and obesity experienced greater symptom reductions than those with healthy weight, despite the fact that their BMI itself did not shift. This is a promising finding, given the strong, well-known association between high weight, poorer mental health and disability. This suggests that the presence of health complexity does not preclude referral to low-cost, accessible internet-based CBT interventions, and that these interventions represent an important adjunct in self-management in the population with overlapping chronic health conditions and obesity or overweight.

## Supplementary information

Supplementary Table 1. Moderator analyses

## Data Availability

Data are available for validation purposes subject to ethical approval from an Australian Human Research Ethics Committee and an appropriate data management agreement.

## References

[1] Australian Institute of Health and Welfare. (2022). Chronic conditions and multimorbidity.

[2] Vos T, Lim SS, Abbafati C, Abbas KM, Abbasi M, Abbasifard M (2020). Global burden of 369 diseases and injuries in 204 countries and territories, 1990–2019: a systematic analysis for the Global Burden of Disease Study 2019. The Lancet..

[3] D’Onghia M, Ciaffi J, Lisi L, Mancarella L, Ricci S, Stefanelli N (2021). Fibromyalgia and obesity: A comprehensive systematic review and meta-analysis. Semin Arthritis Rheum.

[4] Cañete JD, Tasende JAP, Laserna FJR, Castro SG, Queiro R (2020). The Impact of Comorbidity on Patient-Reported Outcomes in Psoriatic Arthritis: A Systematic Literature Review. Rheumatol Ther.

[5] Pearson-Stuttard J, Ezzati M, Gregg EW (2019). Multimorbidity: A defining challenge for health systems. The Lancet Pub Health.

[6] de Ridder DDP, Geenen RP, Kuijer RP, van Middendorp HP (2008). Psychological adjustment to chronic disease. The Lancet (British edition).

[7] Katon WJ (2011). Epidemiology and treatment of depression in patients with chronic medical illness. Dialogues Clin Neurosci.

[8] Roy-Byrne PP, Davidson KW, Kessler RC, Asmundson GJ, Goodwin RD, Kubzansky L (2008). Anxiety disorders and comorbid medical illness. Gen Hosp Psychiatry.

[9] Amiri S, Behnezhad S (2019). Obesity and anxiety symptoms: a systematic review and meta-analysis. Neuropsychiatry.

[10] Luppino FS, de Wit LM, Bouvy PF, Stijnen T, Cuijpers P, Penninx BWJH (2010). Overweight, Obesity, and Depression: A Systematic Review and Meta-analysis of Longitudinal Studies. Arch Gen Psychiatry.

[11] Kyrou I, Osei-Assibey G, Williams N, Thomas R, Halder L, Taheri S (2011). Self-reported disability in adults with severe obesity. J Obes.

[12] Keramat SA, Alam K, Sathi NJ, Gow J, Biddle SJH, Al-Hanawi MK (2021). Self-reported disability and its association with obesity and physical activity in Australian adults: Results from a longitudinal study. SSM Popul Health.

[13] Wadsworth T, Pendergast PM (2014). Obesity (Sometimes) Matters: The Importance of Context in the Relationship between Obesity and Life Satisfaction. J Health Soc Behav.

[14] Department of Health. (2019). National Preventive Health Strategy.

[15] Newman S, Steed L, Mulligan K (2004). Self-management interventions for chronic illness. The Lancet.

[16] Scott A, Bisby M, Heriseanu A, Salameh Y, Karin E, Fogliati R, et al. Cognitive behavioral therapies for depression and anxiety in people with chronic disease: A systematic review and meta-analysis. (Under review). 2022 10.1016/j.cpr.2023.102353 37865080

[17] Mehta S, Peynenburg VA, Hadjistavropoulos HD (2019). Internet-delivered cognitive behaviour therapy for chronic health conditions: a systematic review and meta-analysis. J Behav Med.

[18] Dong HJ, Larsson B, Rivano Fischer M, Gerdle B (2019). Maintenance of quality of life improvement for patients with chronic pain and obesity after interdisciplinary multimodal pain rehabilitation - A study using the Swedish Quality Registry for Pain Rehabilitation. Eur J Pain.

[19] Legha A, Burke DL, Foster NE, van der Windt DA, Quicke JG, Healey EL (2020). Do comorbidities predict pain and function in knee osteoarthritis following an exercise intervention, and do they moderate the effect of exercise? Analyses of data from three randomized controlled trials. Musculoskel Care.

[20] Ma J, Rosas LG, Lv N, Xiao L, Snowden MB, Venditti EM (2019). Effect of integrated behavioral weight loss treatment and problem-solving therapy on body mass index and depressive symptoms among patients with obesity and depression: The RAINBOW randomized clinical trial. JAMA.

[21] Marcus DA. Obesity and the Impact of Chronic Pain. Clin J Pain. 2004;20:186–91. 10.1097/00002508-200405000-00009 15100595

[22] Vitaloni M, Botto-van Bemden A, Sciortino Contreras RM, Scotton D, Bibas M, Quintero M (2019). Global management of patients with knee osteoarthritis begins with quality of life assessment: a systematic review. BMC Musculoskelet Disord.

[23] Flores S, Brown A, Adeoye S, Jason LA, Evans M (2013). Examining the impact of obesity on individuals with chronic fatigue syndrome. Workplace Health Saf.

[24] Jantaratnotai N, Mosikanon K, Lee Y, McIntyre RS (2017). The interface of depression and obesity. Obes Res Clin Pract.

[25] Grigolon RB, Trevizol AP, Gerchman F, Bambokian AD, Magee T, McIntyre RS (2021). Is Obesity A Determinant Of Success With Pharmacological Treatment For Depression? A Systematic Review, Meta-Analysis And Meta-Regression. J Affect Disord.

[26] Dear B, Scott A, Fogliati R, Gandy M, Karin E, Dudeney J (2021). The Chronic Conditions Course: A randomised controlled trial of a remotely-delivered transdiagnostic psychological intervention for people with chronic health conditions. Psychother Psychosom.

[27] Hodge JM, Shah R, McCullough ML, Gapstur SM, Patel AV (2020). Validation of self-reported height and weight in a large, nationwide cohort of U.S. adults. PLOS ONE.

[28] Kroenke K, Spitzer RL, Williams JB (2001). The PHQ-9: validity of a brief depression severity measure. J Gen Intern Med.

[29] Gilbody S, Richards D, Brealey S, Hewitt C (2007). Screening for depression in medical settings with the Patient Health Questionnaire (PHQ): a diagnostic meta-analysis. J Gen Intern Med.

[30] Spitzer RL, Kroenke K, Williams JB, Löwe B (2006). A brief measure for assessing generalized anxiety disorder: the GAD-7. Arch Intern Med.

[31] Kroenke K, Spitzer RL, Williams JB, Monahan PO, Löwe B (2007). Anxiety disorders in primary care: prevalence, impairment, comorbidity, and detection. Ann Intern Med.

[32] Ustün TB, Chatterji S, Kostanjsek N, Rehm J, Kennedy C, Epping-Jordan J (2010). Developing the World Health Organization Disability Assessment Schedule 2.0. Bull World Health Organ.

[33] Garin O, Ayuso-Mateos JL, Almansa J, Nieto M, Chatterji S, Vilagut G (2010). Validation of the “World Health Organization Disability Assessment Schedule, WHODAS-2” in patients with chronic diseases. Health Qual Life Outcomes.

[34] Diener E, Emmons RA, Larsen RJ, Griffin S (1985). The Satisfaction With Life Scale. J Pers Assess.

[35] Karin E, Dear BF, Heller GZ, Crane MF, Titov N (2018). “Wish You Were Here”: Examining Characteristics, Outcomes, and Statistical Solutions for Missing Cases in Web-Based Psychotherapeutic Trials. JMIR Ment Health.

[36] Karin E, Dear BF, Heller GZ, Gandy M, Titov N (2018). Measurement of Symptom Change Following Web-Based Psychotherapy: Statistical Characteristics and Analytical Methods for Measuring and Interpreting Change. JMIR Ment Health.

[37] Hubbard AE, Ahern J, Fleischer NL, Van der Laan M, Lippman SA, Jewell N (2010). To GEE or not to GEE: comparing population average and mixed models for estimating the associations between neighborhood risk factors and health. Epidemiology.

[38] Australian Bureau of Statistics. Overweight and obesity. Australian Bureau of Statistics; 2018.

[39] Austin PC, Jembere N, Chiu M (2018). Propensity score matching and complex surveys. Stat Methods Med Res.

[40] Karin E (2019). Generating and interpreting evidence from psychotherapy: An examination of measurement models, missing cases, and classification methods.

[41] von Elm E, Altman DG, Egger M, Pocock SJ, Gøtzsche PC, Vandenbroucke JP (2007). Strengthening the reporting of observational studies in epidemiology (STROBE) statement: guidelines for reporting observational studies. BMJ.

[42] Thabane L, Mbuagbaw L, Zhang S, Samaan Z, Marcucci M, Ye C (2013). A tutorial on sensitivity analyses in clinical trials: the what, why, when and how. BMC Medical Research Methodology.

[43] Spahlholz J, Baer N, König HH, Riedel-Heller SG, Luck-Sikorski C (2016). Obesity and discrimination – a systematic review and meta-analysis of observational studies. Obes Rev.

[44] Pearl RL, Hopkins CH, Berkowitz RI, Wadden TA (2018). Group cognitive-behavioral treatment for internalized weight stigma: a pilot study. Eat Weight Disord.

[45] Vissers W, Keijsers GPJ, Kampman M, Hendriks GJ, Rijnders P, Hutschemaekers GJM (2017). Symptom Reduction Without Remoralization: A Randomized, Waiting-List Controlled Study Aimed at Separating Two Beneficial Psychotherapy Outcome Effects. J Clin Psychol.

[46] Burki T (2021). European Commission classifies obesity as a chronic disease. The Lancet Diabetes & Endocrinology.

[47] American Psychiatric Association. (2022). Diagnostic and Statistical Manual of Mental Disorders: DSM-5-TR.

[48] Udo T, Grilo CM (2019). Psychiatric and medical correlates of DSM-5 eating disorders in a nationally representative sample of adults in the United States. Int J Eat Disord.

[49] O’Loughlin I, Newton-John TRO (2019). ‘Dis-comfort eating’: An investigation into the use of food as a coping strategy for the management of chronic pain. Appetite.

[50] Satherley R, Howard R, Higgs S (2015). Disordered eating practices in gastrointestinal disorders. Appetite.

[51] Kivimäki M, Strandberg T, Pentti J, Nyberg ST, Frank P, Jokela M (2022). Body-mass index and risk of obesity-related complex multimorbidity: an observational multicohort study. The Lancet Diabetes & Endocrinology.

[52] Faulconbridge LF, Driscoll CFB, Hopkins CM, Bailer Benforado B, Bishop-Gilyard C, Carvajal R (2018). Combined Treatment for Obesity and Depression: A Pilot Study. Obesity (Silver Spring).

[53] Hamer O, Larkin D, Relph N, Dey P (2021). Fear-related barriers to physical activity among adults with overweight and obesity: A narrative synthesis scoping review. Obes Rev.

[54] Sumlin LL, Garcia TJ, Brown SA, Winter MA, García AA, Brown A (2014). Depression and adherence to lifestyle changes in type 2 diabetes: a systematic review. Diabetes Educ.

[55] Burgess E, Hassmén P, Welvaert M, Pumpa KL (2017). Behavioural treatment strategies improve adherence to lifestyle intervention programmes in adults with obesity: a systematic review and meta-analysis. Clin Obes.

[56] Schmidt CB, van Loon BJP, Vergouwen ACM, Snoek FJ, Honig A Systematic review and meta-analysis of psychological interventions in people with diabetes and elevated diabetes-distress. Diabet Med. 2018. 10.1111/dme.13709 10.1111/dme.13709 29896760

[57] You Q, Jiang Q, Li D, Wang T, Wang S, Cao S (2022). Waist circumference, waist-hip ratio, body fat rate, total body fat mass and risk of low back pain: a systematic review and meta-analysis. Eur Spine J.

[58] Sharma AM, Kushner RF (2009). A proposed clinical staging system for obesity. Int J Obes (Lond).

[59] Sebo P, Herrmann FR, Haller DM (2017). Accuracy of anthropometric measurements by general practitioners in overweight and obese patients. BMC Obesity.

